# Passage of an Iron Rod through the Head

**Published:** 1849

**Authors:** J. M. Harlow

**Affiliations:** Cavendish, Vt.


### Part 3.—Selections.

### ARTICLE I.

### *Passage of an Iron Rod through the Head.*

*To the Editor of the Boston Med. Surg. Journal:*

DEAR SIR—Having been interested in the reading of the cases of "Injuries of the Head," reported in your Journal by Professor Shipman, of Cortlandville, N. Y., I am induced to offer you the notes of a very severe, singular, and, so far as the result is taken into account, hitherto unparalleled case, of that class of injuries, which has recently fallen under my own care. The accident happened in this town, upon the line of the Rutland and Burlington Railroad, on the 13th of September last, at 4½ o'clock, P. M. The subject of it is Phineas P. Gage, a foreman, engaged in building the road, 25 years of age, of middle stature, vigorous physical organization, temperate habits, and possessed of considerable energy of character.

It appears from his own account, and that of the bystanders, that he was engaged in charging a hole, preparatory to blasting. He had turned in the powder, and was in the act of tamping it slightly before pouring on the sand. He had struck the powder, and while about to strike it again, turned his head to look after his men (who were working within a few feet of him,) when the tamping iron came in contact with the rock, and the powder exploded, driving the iron against the left side of the face, immediately anterior to the angle of the inferior maxillary bone. Taking a direction upward and backward toward the median line, it penetrated the integuments, the masseter and temporal muscles, passed under the zygomatic arch, and (probably) fracturing the temporal portion of the sphenoid bone, and the floor of the orbit of the left eye entered the cranium. passing through the anterior left lobe of the cerebrum, and made its exit in the median line, at the junction of the coronal and sagittal sutures, lacerating the longitudinal sinus, fracturing the parietal and frontal bones extensively, breaking up considerable portions of brain, and protruding the globe of the left eye from its socket by nearly one half its diameter. The tamping iron is round, and rendered comparatively smooth by use. It is pointed at the end which entered first, and is three feet seven inches in length, one and a quarter inch in diameter, and weighs 13¼ pounds. I am informed that the patient was thrown upon his back, and gave a few convulsive motions of the extremities, but spoke in a few minutes. His men (with whom he was a great favorite,) took him in their arms and carried him to the road, only a few rods distant, and sat him into an ox cart, in which he rode, sitting erect, full three quarters of a mile, to the hotel of Mr. Joseph Adams, in this village. He got out of the cart himself, and, with a little assistance, walked up a long flight of stairs, into the hall, where he was dressed.

Being absent, I did not arrive at the scene of the accident until near 6 o'clock, P. M. You will excuse me for remarking here, that the picture presented was, to one unaccustomed to military surgery, truly terrific; but the patient bore his sufferings with the most heroic firmness. He recognized me at once, and said he hoped he was not much hurt. He seemed to be perfectly conscious, but was getting exhausted from the hemorrhage, which was very profuse both externally and internally, the blood finding its way into the stomach, which rejected it as often as every 15 or 20 minutes. Pulse 60, and regular. His person, and the bed on which he was laid, were literally one gore of blood. Assisted by my friend, Dr. Wilson, of Proctorsville, who was first called to the patient, we proceeded to dress the wounds. From their appearance, the fragments of bone being uplifted and the brain protruding, it was evident that the fracture was occasioned by some force acting from below upward. The scalp was shaven, the coagula removed, together with three small triangular pieces of the cranium; and in searching to ascertain if there were other foreign bodies there, I passed in the index finger its whole length, without the least resistance, in the direction of the wound in the cheek, which received the other finger in like manner. A portion of the anterior superior angle of each parietal bone, and a semi-circular piece of the frontal bone, were fractured, leaving a circular opening of about 2½ inches in diameter. This examination, and the appearance of the iron, which was found some rods distant smeared with brain, together with the testimony of the workmen, and of the patient himself, who was still sufficiently conscious to say that "the iron struck his head and passed through," was considered at the time sufficiently conclusive to show not only the nature of the accident, but the manner in which it occurred.

I have been asked why I did not pass a probe through the entire extent of the wound at the time. I think no surgeon of discretion would have upheld me in the trial of such a foolhardy experiment, in the risk of disturbing lacerated vessels, from which the hemorrhage was near being staunched, and thereby rupturing the attenuated thread by which the sufferer still held to life. You will excuse me for being thus particular, inasmuch as I am aware that the nature of the injury has been seriously questioned by many medical men for whom I entertain a very high respect.

The spiculæ of bone having been taken away, a portion of the brain, which hung by a pedicle, was removed, the larger pieces of bone replaced, the lacerated scalp was brought together as nearly as possible, and retained by adhesive straps, excepting at the posterior angle, and over this a simple dressing—compress, night-cap, and roller. The wound in the face was left patulous, covered only by a simple dressing. The hands and fore arms were both deeply burned nearly to the elbows, which were dressed, and the patient was left with the head elevated, and the attendants requested to keep him in that position.

10 P. M., same evening.—The dressings are saturated with blood, but the hemorrhage appears to be abating. Has vomited twice only since being dressed. Sensorial powers remain as yet unimpaired. Says he does not wish to see his friends, as he shall be at work in a day or two. Tells where they live, their names, etc. Pulse 65; Constant agitation of the lower extremities.

14th, 7, A. M.—Has slept some; appears to be in pain; speaks with difficulty; tumefaction of face considerable, and increasing; pulse 70; knows his friends and is rational. Asks who is foreman in his pit. Hemorrhage internally continues slightly. Has not vomited since 12 M.

15th, 9 A. M.—Has slept well half the night. Sees objects indistinctly with the left eye when the lids are separated. Hemorrhage has ceased. Pulse 75.

8 P. M., same day.—Restless and delirious; talks much, but disconnectedly and incoherently. Pulse 84, and full. Prescribed vin. colchicum, f ʒss every six hours until it purges him. Removed the night cap.

16th, 8 A. M.—Patient appears mora quiet. Pulse 70. Dressed the wounds, which in the head have a fœtid sero-purulent discharge with particles of brain intermingled. No discharge from the bowels. Ordered sulph. magnesia, ℥j, repeated every four hours until it operates. Iced water to the head and eye. A fungus appears at the external canthus of the left eye. Says "the left side of his head is banked up."

17th, 8 P. M.—Pulse 84. Purged freely. Rational, and knows friends. Discharge from the brain profuse, very fœtid sanious. Wound in face healing.

18th, 9, A. M.—Slept well all night, and lies upon his right side. Pulse 72; tongue red and dry; breath fœtid. Removed the dressings, and passed a probe to the base of the cranium, without giving pain. Ordered a cathartic, which operated freely. Cold to the head. Patient says he shall recover. He is delirious, with lucid intervals.

19th, 7 P. M.---Has been very restless during the day, skin hot and dry; tongue red; exessive thirst; delirious, talking incoherently with himself, and directing his men.

20th and 21st.---Has remained much the same.

22nd, 8 A. M.---Patient has had a very restless night. Throws his hands and feet about, and tries to get out of bed. Head hot. Says "he shall not live long so." Ordered a cathartic of calomel and rhubard, to be followed by castor oil, if it does not operate in six hours.

4 P. M. same day.---Purged freely twice and inclines to sleep.

23d.---Rested well most of the night and appears stronger and more rational. Pulse 80. Shaved the scalp a second time, and brought the edges of the wound in position, the previous edges have sloughed away. Discharge less in quantity and less fœtid. Loss of vision of left eye.

From this time till 3d of October, he lay in a semi-comatose state, seldom speaking unless spoken to, and then answering only in monosyllables. During this period, fungi started from the brain, and increased rapidly from the orbit. To these were applied nitrate of silver cryst., and cold to the head generally. The dressings were renewed three times in every twenty-four hours; and in addition to this, laxatives combined with an occasional dose of calomel, constituted the treatment. The pulse varied from 70 to 96---generally very soft. During this time an abscess formed under the frontalis muscle, which was opened on the 27th, and has been very difficult to heal. Discharged nearly ℥viij at the time it was punctured.

Oct. 5th and 6th.---Patient improving. Discharge from the wound and sinus, laudable pus. Calls for his pants and wishes to get out of bed, though he is unable to raise his head from the pillow.

7th.—Has succeeded in raising himself up, and took one step to his chair and sat down about five minutes.

11th.—Pulse 72. Intellectual faculties brightening. When I asked him how long since he was injured, he replied "four weeks this afternoon, at 4½ o'clock. Relates the manner in which it occurred, and how he came to the house. He keeps the day of the week and time of day, in his mind. Says he knows more than half of those who inquire after him. Does not estimate size or money accurately, though he has memory as perfect as ever. He would not take $1000 for a few pebbles which he took from an ancient river bed where he was at work. The fungus is giving way under the use of the crys. nitrate of silver. During all this time there has been a discharge of pus into the fauces, a part of which passed into the stomach, the remainder being ejected from the mouth.

20th.—Improving. Gets out and into bed with but little assistance. Sits up thirty minutes twice in twenty-four hours. Is very childish; wishes to go home to Lebanon, N. H. The wound in the scalp is healing rapidly.

Nov. 8th.—Improving in every particular, snd sits up most of the time during the day. Appetite good, though he is still kept upon a low diet. Pulse 65. Sleeps well, and says he has no pain in the head. Food digests easily, bowels regular and nutrition is going on well. The sinus under the frontalis muscle has nearly healed. He walks up and down stairs, and about the house, into the piazza, and I am informed this evening that he has been in the street to-day. I leave him for a week, with strict injunctions to avoid excitement and exposure.

15th.—I learn on inquiry, that Gage has been in the street every day except Sunday, during my absence. His desire to be out and to go home to Lebanon has been uncontrollable by his friends, and he has been making arrangements to that effect. Yesterday he walked half a mile, and purchased some small articles at the store. The atmosphere was cold and damp, the ground wet, and he went without an overcoat and with thin boots. He got wet feet and a chill. I find him in bed, depressed, and very irritable. Hot and dry skin; thirst; tongue, coated; pulse 110; lancinating pain in left side of head and face; rigors, and bowels constipated. Ordered cold to the head and face, and a black dose to be repeated in six hours, if it does not operate. He has had spiculæ of bone pass into the fauces, which he expelled from the mouth within a few days.

16th, A. M.---No better. Cathartic has operated freely. Pulse 120; skin hot and dry; thirst and pain remain the same. Has been very restless during the night. Venesec. f ℥xvj. Ordered calomel grs. x, and ipecac. grs. ij, followed in four hours by castor oil.

8 P. M. same day.---Purged freely; pulse less frequent; pain in head moderated; skin moist. ℞. Antim. et potassa tart. grs. iij; syr. simplex, f ℥vj. Dose a dessert spoonful every four hours.

17th.---Improving. Expresses himself as feeling better in every respect; has no pain in the head.

18th.---Is walking about the house again; says he feels no pain in the head, and appears to be in a way of recovering if he can be controlled.

At this date I shall leave the case at present. The result, and a few remarks of a practical nature, together with the mental manifestations of the patient, I reserve for a future communication. I think the case presents one fact of great interest to the practical surgeon, and, taken as a whole, is exceedingly interesting to the enlightened physiologist and intellectual philosopher. In my effort to be brief, which I fear you will think an utter failure, I have omitted much in my notes that might interest some readers. Allow me to say here, that I have seen a communication in "The Reflector and Watchman," stating that "there is a piece of bone loose in the top of his head, as large as a dollar, which will have to be removed should he live." The fractured portions of bone, excepting those which were removed at the first dressing, have united firmly, and the above remark was made unadvisedly. Should you think these notes of sufficient importance to deserve a place in your Journal, they are at your service.

Yours, very respectfully,

*Cavendish*, *Vt.*, *Nov.* 27, 1848. J. M. HARLOW.

